# The Impact of Ambient Temperature on Cardiorespiratory Mortality in Northern Greece

**DOI:** 10.3390/ijerph20010555

**Published:** 2022-12-29

**Authors:** Kyriaki Psistaki, Ioannis M. Dokas, Anastasia K. Paschalidou

**Affiliations:** 1 Department of Forestry and Management of the Environment and Natural Resources, Democritus University of Thrace, 68200 Orestiada, Greece; 2 Department of Civil Engineering, Democritus University of Thrace, 67100 Xanthi, Greece

**Keywords:** temperature, cardiorespiratory mortality, time-series, relative risk, attributable risk, Mediterranean region

## Abstract

It is well-established that exposure to non-optimum temperatures adversely affects public health, with the negative impact varying with latitude, as well as various climatic and population characteristics. This work aims to assess the relationship between ambient temperature and mortality from cardiorespiratory diseases in Eastern Macedonia and Thrace, in Northern Greece. For this, a standard time-series over-dispersed Poisson regression was fit, along with a distributed lag nonlinear model (DLNM), using a maximum lag of 21 days, to capture the non-linear and delayed temperature-related effects. A U-shaped relationship was found between temperature and cardiorespiratory mortality for the overall population and various subgroups and the minimum mortality temperature was observed around the 65th percentile of the temperature distribution. Exposure to extremely high temperatures was found to put the highest risk of cardiorespiratory mortality in all cases, except for females which were found to be more sensitive to extreme cold. It is remarkable that the highest burden of temperature-related mortality was attributed to moderate temperatures and primarily to moderate cold. The elderly were found to be particularly susceptible to both cold and hot thermal stress. These results provide new evidence on the health response of the population to low and high temperatures and could be useful to local authorities and policy-makers for developing interventions and prevention strategies for reducing the adverse impact of ambient temperature.

## 1. Introduction

As human health is inextricably linked to the quality of life, well-being, and economic growth [1] and under the threat of a rapidly changing climate, the relationship between public health and weather conditions has been a burning issue for the scientific community during the last decades. A large body of the international literature has associated cold spells, heat waves, and extreme temperatures with increased morbidity and mortality worldwide e.g., [2,3,4,5,6,7,8,9]. One of the most remarkable examples in Europe was the fatal heat wave during the summer of 2003, which cost the life of 80,000 people [10]. Besides extreme temperatures, exposure to moderately high or low temperatures has been found to negatively affect public health e.g., [11,12,13,14,15]. In addition, recent studies demonstrated the adverse health effects of variable temperature, using indicators such as the diurnal temperature range [16,17,18,19] and the temperature variability (i.e., the standard deviation of minimum and maximum temperatures during a given time period) [20,21].

The relationship between temperature and mortality is nonlinear, usually in the form of a “U”, “V” or “J” curve, where the minima of the curve correspond to the temperature (or temperature range) where mortality is minimized, so-called minimum mortality temperature (MMT) e.g., [4,15,22,23,24]. It is well-established that high temperatures have an almost direct effect on health which lasts a few days, while the impact of cold can be observed up to a month after the exposure [13,15,23]. On the whole, non-optimum temperatures affect the function of the thermoregulatory system, triggering different physiological mechanisms [25] which may result in morbidity or mortality from a wide range of causes, including cardiovascular (i.e., myocardial infarction or stroke) and respiratory (i.e., chronic obstructive pulmonary disease) diseases, diabetes, as well as genitourinary and neurological disorders (i.e., Alzheimer’s disease and dementia) [26,27,28,29,30,31]. In addition, low temperatures can act synergistically with various factors favoring respiratory infections from viruses such as influenza, respiratory syncytial virus (RSV), and human parainfluenza virus type 2 (HPIV-2) [32].

The severity of cold- or heat-related health effects depends on many factors, including latitude, demographic and socioeconomic characteristics, housing, air-conditioning use, and acclimatization via behavioral changes, such as proper clothing and exercise [22,33,34,35,36,37]. The elderly and people with underlying medical conditions, as well as young children and pregnant women, have been identified as the most susceptible groups of the population [2,37,38,39,40,41,42]. In addition, differences have been observed in temperature-related vulnerability between genders [15,43]. Several studies have demonstrated that people with low socioeconomic status and income are particularly prone to extreme temperatures, probably because they usually live in poor-quality housing, receive insufficient medical care, and lack access to air conditioning [33,37,39,44]. Moreover, the regional climate is an important factor affecting population tolerance to thermal stress, with residents of relatively cold or hot regions being more vulnerable to heat and cold effects, respectively [23,45,46].

Although the ongoing global warming trend is expected to result in increased heat-related mortality in the future [47,48,49], there is some evidence of population adaptation to high temperatures [36,37,50,51,52], while a concomitant maladaptation to low temperatures has also been observed [36,46,53]. Considering the complexity of cold-related health effects, as well as the finding that low temperatures usually impose a greater risk of mortality [8,22,54,55,56,57], the examination of human response to all temperature ranges remains crucial.

This study aims to explore the impact of both high and low ambient temperatures on mortality from cardiorespiratory diseases in a sub-region of the Mediterranean basin, the region of Eastern Macedonia, and Thrace (EMT) in the northeastern part of Greece. The location of the Mediterranean basin along with various climatological and socioeconomic factors make it one of the most responsive to climate change regions in the world [58]. Specifically, the Mediterranean basin already experiences 1.5 °C higher surface temperature compared to pre-industrial times and future projections demonstrate steadily increasing temperatures, more intense, frequent, and long heat waves, as well as a decrease, but no elimination, of cold spells [37,59,60]. Regarding Greece, the vast majority of existing literature is focused on the two largest urban centers, Athens and Thessaloniki e.g., [4,61,62,63,64,65,66,67,68]. However, the impact of thermal stress has not been explored in other parts of the country. EMT is of special interest, as it is the poorest region of Greece, featuring the lowest per capita income and a slightly higher rate of the elderly population (21.4%) compared to the national (19.5%) (Hellenic Statistical Authority, census 2011) and the European average (20.8%) (Eurostat). Therefore, it provides a unique opportunity to study the impact of thermal stress on a more aged population with a rather low socio-economic status.

## 2. Materials & Methods

### 2.1. Area Description

**Figure 1:**
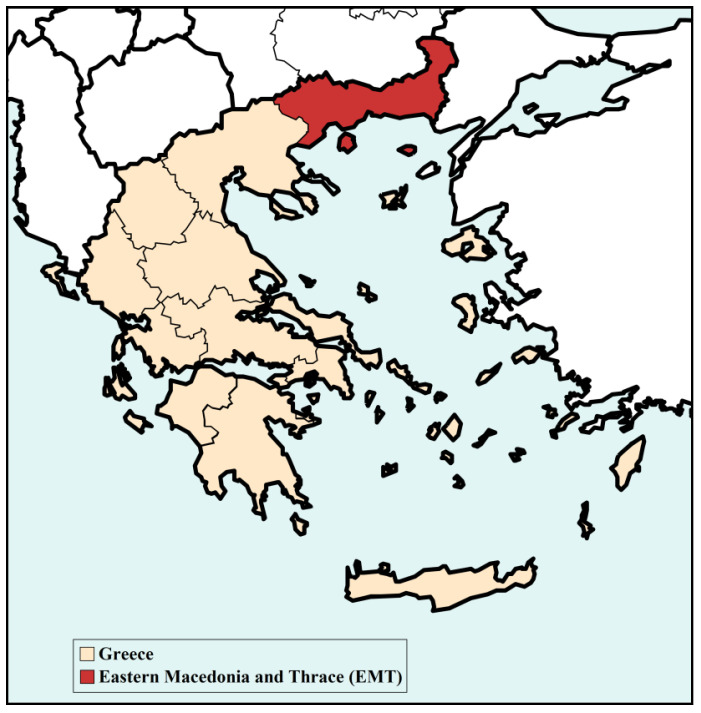
The region of Eastern Macedonia and Thrace (EMT) is colored in red.

EMT is one of the thirteen first-level administrative entities of Greece, extending at an area of over 14,157 km² in the northeastern part of the country and bordering Turkey to the East, Bulgaria to the North, and the Aegean Sea to the South (Figure 1). ΕΜΤ combines the coastal region on the south with mountainous areas on the north and extensive flatlands, mainly in the central and southern parts of the region. The highest peaks are Mount Falakro (2232 m) and Orvilos (2212 m), both located in the regional unit of Drama. Moreover, two rivers cross the region, namely river Nestos at the central part of the region and river Evros which is a natural border for Greece, Bulgaria, and Turkey.

EMT has a Mediterranean climate characterized by hot, dry summers and mild, wet winters. The aforementioned geomorphological diversity contributes to the climatic variability observed between the coastal areas and the mainland. In the latter, lower temperatures and snowfall are usually observed during winter.

According to the census of 2021 (Hellenic Statistical Authority; https://www.statistics.gr/statistics/pop (accessed on 21 December 2022)), the total population of the region is 562,069, of which 51% are females. In terms of the populace, the largest cities in EMT are Alexandroupolis (72,959 residents), Xanthi (66,162), Kavala (65,857 residents), Komotini (65,107), Drama (55,593), and Orestiada (37,695 residents). One-fifth of the population in EMT has an age of 65 years and over and this region has the lowest per capita income in Greece

### 2.2. Data and Methods

Daily meteorological and mortality data from 1999 to 2018 were used for this study. The mean daily values of temperature (°C) and relative humidity (%) were averaged over data collected in three meteorological stations (Table S1). The daily cardiorespiratory mortality was estimated as the sum of daily mortality from respiratory (ICD-10 code: J00-J99) and cardiovascular (ICD-10 code: I00-I99) diseases, obtained from the Hellenic Statistical Authority.

To evaluate the non-linear and lagged effects of daily mean temperature on cardiorespiratory mortality, a standard time-series over-dispersed Poisson regression model was fit, coupled with a distributed lag nonlinear model (DLNM) [69,70]. A maximum lag of 21 days was used to capture the delay in cold-related effects and to adjust for possible temporary displacement of mortality (harvesting effect) [22,23,71,72]. The temperature-mortality relationship and the lagged effect were modeled using a natural cubic spline function with 3 knots, placed at equally-spaced values in the temperature range and in the log scale of lags, to allow enough flexibility [69,70]. To control for long-term trends and seasonality, the model included a natural cubic spline of time, with 8 degrees of freedom per year, based on the minimization of Akaike’s information criterion for overdispersed data. In addition, a natural cubic spline for relative humidity with 3 degrees of freedom and a categorical variable for the day of the week were used as additional confounders.

Based on the aforementioned models and centering at the median value of mean daily temperature e.g., [22], the minimum mortality temperature (MMT) corresponding to the lowest risk of cardiorespiratory mortality, as well as the corresponding minimum mortality percentile (MMP), were estimated. The MMT represents the threshold below (or above) which mortality increases.

Then, to assess the exposure-response relationship between temperature and health effects, the cumulative relative risk of cardiorespiratory mortality was estimated for an overall period of 21 days (lag 0–21) and specific lags (lag 0, lag 1–2, lag 3–5, lag 6–21) at extreme and moderate temperatures defined at the 1st (extreme cold), 10th (moderate cold), 90th (moderate heat) and 99th (extreme heat) percentile of the temperature distribution. MMT was set as the reference value and the temperature was considered steady during the whole lag period examined (cumulative risk).

Although the relative risk RR is widely used, it often proves inadequate to capture the magnitude of temperature-related health impact, as high RR does not necessarily coincide with a high number of casualties e.g., [15,22]. To overcome this issue, the total number of deaths (AN) and the fraction of mortality (AF) attributed to non-optimum temperatures were also estimated for exposure to moderate cold/heat (temperatures between MMT and the 1st percentile/temperatures between MMT and the 99th percentile) and extreme cold/heat (temperatures lower than the 1st percentile/temperatures higher than the 99th percentile) using the backward estimation approach [73]. Empirical confidence intervals (eCI) were calculated for AF at 95%, using 1000 Monte Carlo simulations and assuming a multivariate normal distribution defined by the original parameter estimates and their covariance matrix [73].

Finally, a sensitivity analysis was conducted by changing the df for the time variable and relative humidity, using different maximum lag days for the temperature-mortality association and without controlling for relative humidity. The statistical tests were two-sided with a 0.05 level of significance. All statistical analyses described above were conducted separately for the overall population and various subgroups (males, females, elderly).

## 3. Results

Between 1999 and 2018, 72,123 people died from cardiorespiratory diseases in EMT. females had a higher death rate (52.1%) than males, while 90% of deaths were among the population aged 65 years old and over. On average, the daily cardiorespiratory mortality was 9.87 (SD: 3.50) for the overall population, 4.73 (SD: 2.28) for males, 5.15 (SD: 2.43) for females, and 8.92 (SD: 3.32) for the elderly (Table 1). The daily mean, maximum and minimum temperatures followed a slightly increasing trend throughout the years (not shown), with average values equal to 16.2 °C (SD: 8.36 °C), 18.23 °C (SD: 8.39 °C) and 11.89 °C (SD: 7.77 °C), respectively (Table 1).

**Figure 2:**
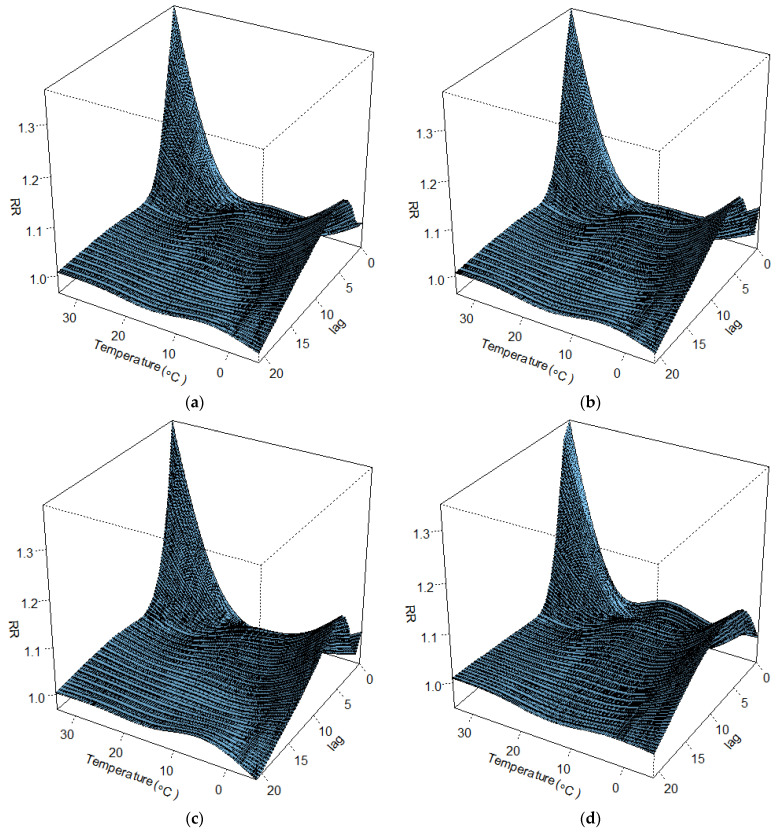
3D plots of the non-linear relationship between mean temperature and cardiorespiratory mortality in EMT between 1999 and 2018 for (**a**) the total population, (**b**) the elderly, (**c**) males, and (**d**) females.

**Figure 3:**
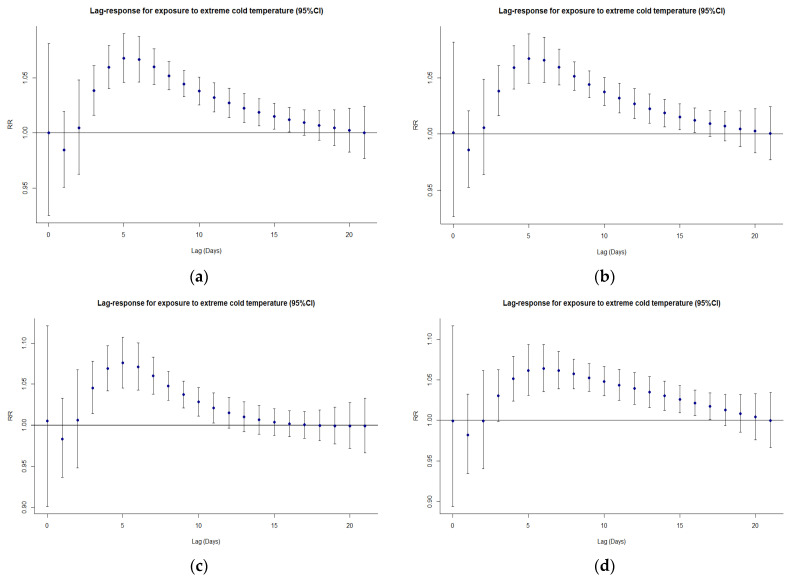
Lag-response curves for exposure to extreme cold (95% CI) for (**a**) the total population, (**b**) the elderly, (**c**) males, and (**d**) females.

**Figure 4:**
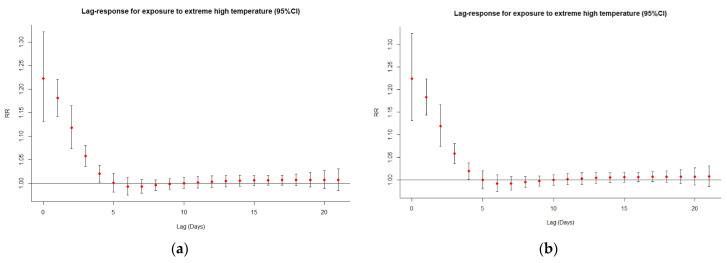

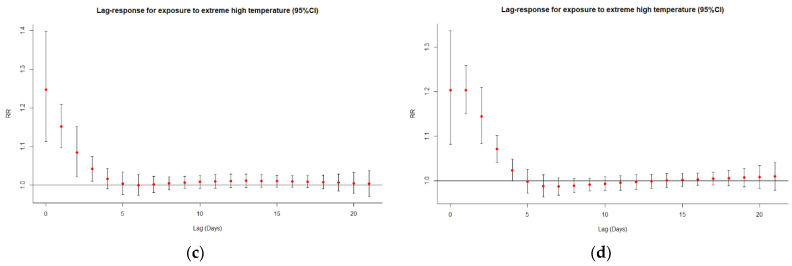
Lag-response curves for exposure to extreme heat (95% CI) for (**a**) the total population, (**b**) the elderly, (**c**) males, and (**d**) females.

Figure 2 illustrates the relative risks of cardiorespiratory deaths, highlighting the non-linear and delayed effects (21 days) of temperature on mortality. The exposure-response curve of extreme cold for the overall population, males, and the elderly peaked at lag 5 and then decreased gradually (Figure 3), as opposed to the exposure-response curve of extreme heat which peaked around lag 0 and decreased steeply afterward (Figure 4). Similar patterns were observed for females, although the peaks for extreme cold and heat were found on lag 6 and lag 1, respectively (Figure 3 and Figure 4). Moreover, in all cases (except for males) the relative risk of cardiorespiratory mortality due to extremely high temperatures dropped below 1 between lag 5 and lag 10 approximately, indicating a suggestive harvesting effect (Figure 4). The analysis of exposure to moderate temperatures revealed similar trends (Figures S1 and S2).

**Figure 5:**
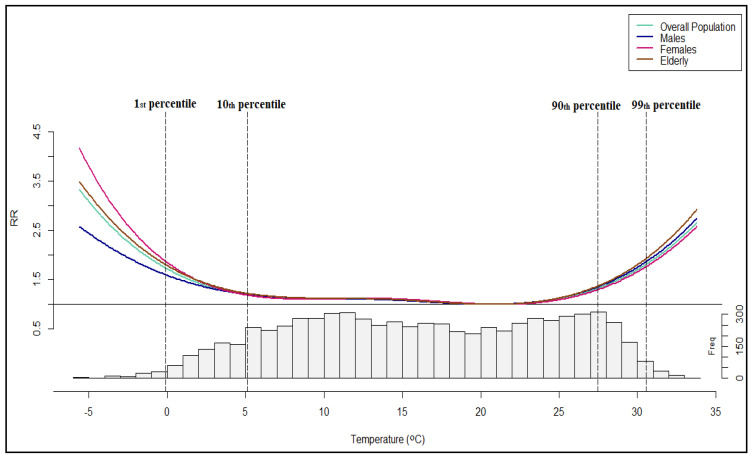
The cumulative exposure-response curve of mean daily temperature for the total population and its subgroups in EMT for a lag period of 21 days.

ΜΜΤ was observed at 4.7 °C above the average mean daily temperature, at the 65th percentile of the temperature distribution (20.9 °C) for the overall population and the elderly, at the 64th percentile (20.5 °C) for males and the 67th percentile (21.4 °C) for females (Table 2). In all cases, the cumulative exposure-response relationship between mean temperature and cardiorespiratory mortality was depicted by a U-shaped curve, where the lowest extrema corresponded to MMT (Figure 5). A closer look at Figure 5 reveals significant gender differences in the mortality risk for temperatures below the 1st percentile, where it is obvious that females are the most susceptible group of the population to extreme cold, followed by the elderly. However, such extreme temperatures rarely occur in EMT, as they comprise only 1.08% of the total number of days (Figure 5, Table S2).

As shown in Table 2, when considering the whole period of 21 days, the highest overall relative risks of mortality were estimated for exposure to extreme temperatures. Regarding the two genders, exposure to extreme heat was more dangerous for males (lag 0–21: 1.88, CI: 1.44–2.44), whereas females were found to be more sensitive to extreme cold, with a relative risk of cardiorespiratory mortality equal to 1.88 (CI: 1.44–2.45) on lag 0–21 (Table 2). These gender differences were statistically significant according to the Chi-square test. Moreover, the elderly were found to be particularly vulnerable to temperature-related mortality, with the highest values of overall relative risk estimated under extreme heat (lag 0–21: 1.94, CI: 1.60–2.36) (Table 2). Regarding moderate temperatures, moderately hot conditions were more dangerous for cardiorespiratory mortality in EMT compared to moderate cold. A thorough examination of Table 2 reveals that the cumulative relative risks of high temperatures were maximized at lags 0 and lags 1–2, as opposed to the relative risks of low temperatures that were apparent from lag 3 and onwards.

**Figure 6:**
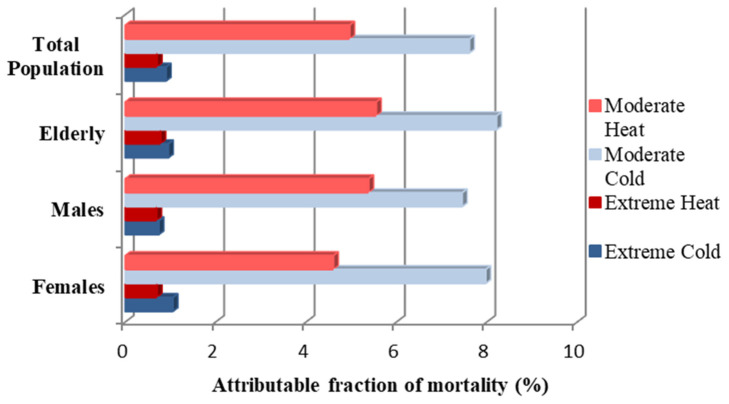
The fraction of mortality attributed to moderate and extreme temperatures for the total population and its subgroups in EMT.

On the whole, from the 72,123 cardiorespiratory deaths recorded in EMT during the period 1999–2018, 10,035 were attributed to non-optimum temperatures. The number of casualties was 9896 for the elderly, 4841 for males, and 5289 for females (Table S2). Figure 6 depicts the fraction of mortality attributed to extreme and moderate temperatures. It is apparent that moderate temperatures were responsible for the highest-burden of cardiorespiratory mortality (Figure 6). The relative figures for moderately low temperatures ranged from 7.45% for males to 8.21% for the elderly, while for moderately high temperatures the figures ranged from 4.6% for females to 5.55% for the elderly (Table S2). Moreover, it should be mentioned that between the two extreme thermal conditions, the extreme cold had a slightly greater impact on mortality (Figure 6, Table S2). All attributable fractions estimated, except for those for the gender-specific mortality under moderately cold conditions, were statistically significant according to the two-sided test (*p*-value < 0.05). Finally, the sensitivity analysis resulted in similar results, indicating that the effects of temperature on cardiorespiratory mortality did not depend on the selection of models (Tables S3 and S4, Figures S3–S8).

## 4. Discussion

This work examined the impact of mean daily temperature on cardiorespiratory mortality for the overall population and various subgroups in the region of EMT in Greece. The exposure-response associations were found non-linear forming U-shaped curves, in accordance with previous studies e.g., [22,23,24,43,66,74]. MMT was defined at the 65th percentile (20.9 °C) of the temperature distribution for the overall population and ranged between the 64th percentile (20.5 °C) for males and the 67th (21.4 °C) percentile for females.

MMT is generally cause-specific and varies across regions following a decreasing trend with latitude [22,23,24,57,66,75] which indicates some population adaptability to the local climate. In recent work, Psistaki et al. (2023) observed a reverse J-shaped relationship between thermal stress and cardiovascular mortality for the overall population in Thessaloniki (Greece) and defined the MMT at 25.4 °C [68]. Furthermore, Kouis et al. (2019) reported that heat-related mortality from respiratory and cardiovascular causes in Thessaloniki starts when the temperature exceeds the threshold of 33 °C [3]. Following a different methodology for Athens (Greece), Dimitriadou et al. (2022) defined thresholds for cold- and heat-related cardiorespiratory mortality at 9.76 °C and 24.23 °C, respectively [66]. Tsoutoubi et al. (2021) found that mortality from circulatory diseases was minimized in the temperature range between 6 °C and 39 °C for the Greek population over 70 years old [76]. Although, a direct comparison of these results is impossible due to the different statistical techniques and exposure variables (e.g., daily maximum temperature, apparent temperature, mean daily temperature) used, the rather small MMT values observed herewith could reflect the higher rate of people at the age of 65+ in EMT and their lower socioeconomic status compared to neighboring regions in Greece [2,37,39,40,77].

The well-established delayed effect of low temperatures and the almost immediate impact of high temperatures [15,23,55,57,74,78] were confirmed in this work, with the risk of cardiorespiratory mortality spiking around lag 5 and lag 0, respectively. Consistent with findings from previous studies [4,15,23,67], a displacement in heat-related mortality was observed a week after exposure, suggesting that high temperatures probably accelerated the death of vulnerable populations who would have died regardless of their exposure to ambient weather conditions.

In agreement with other studies [15,24,43,47,57,67,68,73,79], although the highest relative risks of cardiorespiratory mortality were estimated under extreme temperatures, moderate thermal conditions, and especially moderate cold, caused the highest burden of mortality in EMT. These findings do not come as a surprise, as in our study extremely cold and hot days comprised only 2.04% of the total days. In addition, the population might have been more conscious during extremely cold or hot days, perceiving them as more dangerous and minimizing exposure. It is of note that the broad empirical confidence intervals in attributable fractions for moderately cold conditions and the insignificant two-sided test (*p*-value < 0.05) estimated for the gender-specific AFs under this temperature range, might have arisen from the relatively small sample size [15].

Regarding the bigger impact of low temperatures observed herewith, there is evidence that people living in warm regions, like the Mediterranean, tend to be acclimatized to the heat and are therefore less sensitive to heat-related health effects [11,23,37]. Except for the physiological adaptation of the population, these results might have stemmed from public awareness and the effective implementation of preventive measures (e.g., using air conditioning, drinking enough water, and staying indoors) to face high temperatures and heat waves that frequently afflict the Mediterranean region [37,80]. Although the heat-related health impact on populations living in cities may be intensified by poor air quality [81,82,83] and increased temperatures due to the urban heat island effect [33,35,37,39,84], the aforementioned findings could indicate a possible acclimatization of the population to urban climate. The physical and behavioral adaptation to high temperatures has also been reflected in the declining trend of heat-related mortality in the /Mediterranean region throughout the years [36,37]. Nevertheless, considering the increasing trend in intensity, frequency, and duration of heat waves projected for the Mediterranean region [37,59,60], the impact of heat should not be neglected.

Consistent with the existing literature e.g., [3,6,11,19,57,78,85,86,87,88,89,90], this work demonstrated that older people are particularly prone to non-optimum temperatures, which probably stems from the decreasing with age ability of thermoregulation, along with co-existing health problems and socioeconomic factors including low income and isolation [37,91,92,93]. Specifically, our results showed that extremely high and secondarily extremely low temperatures put the highest risk of cardiorespiratory mortality for the elderly, while the largest burden of mortality was attributed to moderate cold. Similarly, Han et al. (2017) demonstrated that the elderly in China were particularly prone to heat waves, while cold spells affected the population aged below 65 years more [94]. On the other hand, some studies have established stronger associations between low temperatures and mortality for the aged population [9,95,96,97]. For instance, Liu et al. (2020) and Yi and Chan (2015) found that low temperatures were more dangerous for public health in Hong Kong and the risk of mortality due to extreme cold increased with age [15,98]. Interestingly, the latter observed that people aged between 65 and 74 years old were afflicted by hot temperatures more than older people.

According to the gender-specific analysis, the highest overall relative risk of mortality was estimated under extreme cold for females and extreme heat for males. However, the highest burden of cardiorespiratory mortality for both genders was attributed to moderate cold. These differences in temperature-related health effects between the two genders could have arisen from physiological characteristics such as the sweating response to heat and body fat, as well as socioeconomic factors [95,99,100]. However, studies are rather inconsistent on this issue [11]. A large number of works have demonstrated that females are more vulnerable to thermal stress e.g., [14,43,78,83,93,101], while others have found more pronounced effects for males e.g., [6,19]. Recent work for Scotland concluded that low temperatures affected males more, while females were more afflicted by high temperatures [90]. Similarly, a study focused on Spain [89] reported a higher risk of heat-related CVD mortality for females, whereas males were found more vulnerable to low temperatures. Nevertheless, it should be noted that some studies observed no significant differences in the vulnerability of the two genders [85,102].

It should be kept in mind that the different statistical approaches used in each study, as well as the differences in population characteristics (e.g., socioeconomic status, lifestyle, age, gender), the local climate, and the health outcome under study, may result in discrepancies among the findings of various epidemiological studies.

## 5. Conclusions

To our knowledge, this study examined for the first time the impact of ambient temperature on cardiorespiratory mortality for the overall population and various subgroups in Eastern Macedonia and Thrace, in Northern Greece. In all cases, the relationship between temperature and mortality depicted a U-shaped curve, with the minimum mortality temperature observed at 20.9 °C. This rather low figure probably reflected the high ratio of the aged population and its lower socioeconomic status, and highlighted the importance of confounding factors, such as the age and socioeconomic parameters on the relationships between ambient temperature and cardiorespiratory mortality.

Our study confirmed the delayed effect of low temperatures and the almost immediate impact of high temperatures, while some evidence of mortality displacement was provided. It was found that the risk of cardiorespiratory mortality increased significantly for exposure to extremely high temperatures, in all cases except for females who comprised the only group of the population being more prone to extreme cold. The elderly were found to be particularly susceptible to cold and hot thermal stress. In all cases, moderate temperatures were responsible for the highest-burden of temperature-related cardiorespiratory mortality, with moderate cold playing the primary role.

These findings could provide useful information to local authorities and policy-makers to develop prevention strategies for reducing the effects of thermal stress on cardiorespiratory mortality, with the emphasis put on the most susceptible groups of the population.

## Figures and Tables

**Table 1:** Descriptive statistics of the daily meteorological variables and daily deaths from cardiorespiratory diseases in EMT from 1999 to 2018.

	Mean	Median	Standard Deviation	Min	Max	1st Percentile	10th Percentile	90th Percentile	99th Percentile
Temperature (°C)	16.2	16.1	8.36	−5.67	33.8	−0.12	5.09	27.5	30.6
Maximum Temperature (°C)	18.23	17.97	8.39	−4.15	35.6	1.63	6.90	29.5	32.8
Minimum Temperature (°C)	11.89	11.97	7.77	−10.33	28.3	−3.97	1.32	22.1	25.1
Relative Humidity (%)	65.1	65.3	14.7	16.7	100	22.3	46.7	83.7	92.5
Mortality from cardiorespiratory diseases									
	Mean	Median	Standard Deviation	Min	Max	1st percentile	10th percentile	90th percentile	99th percentile
Total Population	9.87	10	3.50	0	30	3	6	14	19
Males	4.73	5	2.28	0	17	0	2	8	11
Females	5.15	5	2.43	0	16	1	2	8	12
Elderly (≥65 years)	8.92	9	3.32	0	25	2.04	5	13	18

**Table 2:** MMT and cumulative relative risks of cardiorespiratory mortality under various lag intervals, considering constant exposure, for the total population and its subgroups in EMT between 1999 and 2018.

	Minimum Mortality Temperature (MMT, °C)	Minimum Mortality Percentile (MMP)	Lag Period	Relative Risk for Extreme Cold (95% CI)	Relative Risk for Extreme Heat (95% CI)	Relative Risk for Moderate Cold (95% CI)	Relative Risk for Moderate Heat (95% CI)
Total Population	20.9	65	0–21	1.74 (1.44–2.10)	1.82 (1.52–2.18)	1.19 (1.00–1.42)	1.33 (1.20–1.48)
			0	1.00 (0.93–1.08)	1.22 (1.13–1.32)	0.99 (0.93–1.06)	1.11 (1.06–1.17)
			1–2	0.99 (0.92–1.07)	1.32 (1.23–1.42)	0.95 (0.89–1.01)	1.17 (1.12–1.22)
			3–5	1.17 (1.12–1.24)	1.08 (1.03–1.13)	1.09 (1.04–1.14)	1.02 (0.99–1.05)
			6–21	1.50 (1.30–1.73)	1.04 (0.91–1.19)	1.16 (1.02–1.33)	1.00 (0.92–1.07)
Males	20.5	64	0–21	1.60 (1.23–2.08)	1.88 (1.44–2.44)	1.20 (0.94–1.53)	1.36 (1.17–1.59)
			0	1.01 (0.90–1.12)	1.25 (1.11–1.40)	0.98 (0.89–1.08)	1.14 (1.06–1.22)
			1–2	0.99 (0.89–1.10)	1.25 (1.13–1.39)	0.98 (0.89–1.07)	1.13 (1.06–1.20)
			3–5	1.20 (1.12–1.29)	1.06 (0.99–1.14)	1.12 (1.05–1.19)	1.01 (0.97–1.06)
			6–21	1.34 (1.09–1.64)	1.13 (0.93–1.37)	1.12 (0.93–1.35)	1.05 (0.93–1.17)
Females	21.4	67	0–21	1.88 (1.44–2.45)	1.78 (1.39–2.27)	1.19 (0.92–1.52)	1.30 (1.14–1.50)
			0	1.00 (0.89–1.12)	1.20 (1.08–1.34)	1.01 (0.92–1.11)	1.09 (1.03–1.16)
			1–2	0.98 (0.88–1.09)	1.38 (1.25–1.52)	0.91 (0.83–1.00)	1.20 (1.14–1.27)
			3–5	1.15 (1.07–1.24)	1.09 (1.02–1.17)	1.06 (1.00–1.13)	1.03 (1.00–1.07)
			6–21	1.66 (1.35–2.05)	0.98 (0.82–1.17)	1.21 (1.00–1.47)	0.96 (0.87–1.06)
Elderly (≥65 years)	20.7	65	0–21	1.81 (1.49–2.20)	1.94 (1.60–2.36)	1.22 (1.02–1.47)	1.38 (1.23–1.54)
			0	1.02 (0.94–1.10)	1.23 (1.13–1.34)	1.00 (0.93–1.07)	1.12 (1.06–1.18)
			1–2	0.97 (0.90–1.05)	1.35 (1.25–1.46)	0.94 (0.88–1.00)	1.19 (1.14–1.24)
			3–5	1.18 (1.12–1.24)	1.10 (1.04–1.16)	1.09 (1.04–1.14)	1.03 (1.00–1.06)
			6–21	1.55 (1.34–1.81)	1.07 (0.93–1.23)	1.20 (1.04–1.38)	1.00 (0.93–1.09)

## Data Availability

The authors do not have permission to share the data.

## Supplementary Materials

The following supporting information can be downloaded at: https://www.mdpi.com/article/10.3390/ijerph20010555/s1.
