# Supplementary material for: The Impact of Ambient Temperature on Cardiorespiratory Mortality in Northern Greece
Source: Int J Environ Res Public Health. 2022 Dec 29;20(1):555. doi: 10.3390/ijerph20010555 (PMC9819162; doi:10.3390/ijerph20010555)
Supplement: Supplementary file 1 [file ijerph-20-00555-s001.zip › ijerph-2120217-supplementary.pdf]

Table S1: Characteristics of the 3 meteorological stations used.

	**Station 1**	**Station 2**	**Station 3**
Longitude	25 56 E	24 36 E	24 25 E
Latitude	40 51 N	40 56 N	41 06 N
Elevation (m)	3.5	4.2	86.9
Minimum mean daily temperature (°C)	-6.9	-4.1	-11.2
Maximum mean daily temperature (°C)	34.3	32.5	37.3
Average mean daily temperature (°C)	16.2	16	16.5
Minimum RH (%)	16.4	16.3	16.3
Maximum RH (%)	100	100	100
Minimum wind speed (m/s)	0	0	0
Maximum wind speed (m/s)	20.8	18	37.7

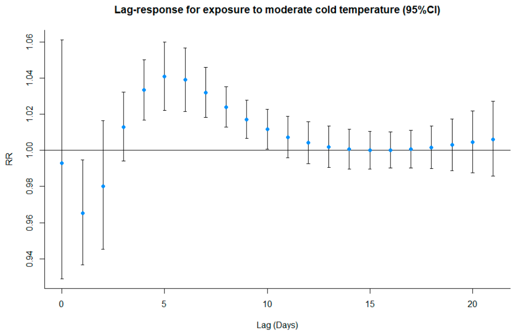

a) Total population

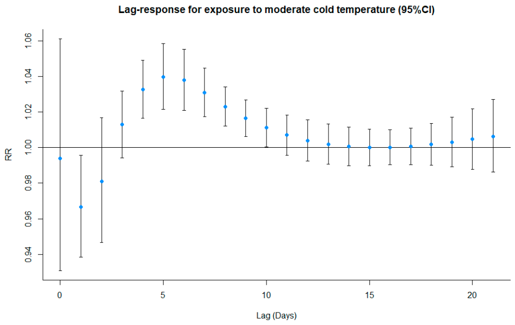

b) Elderly

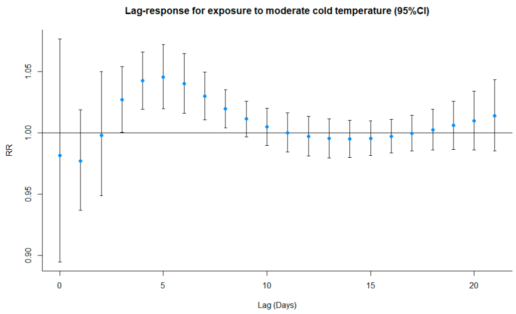

c) Males

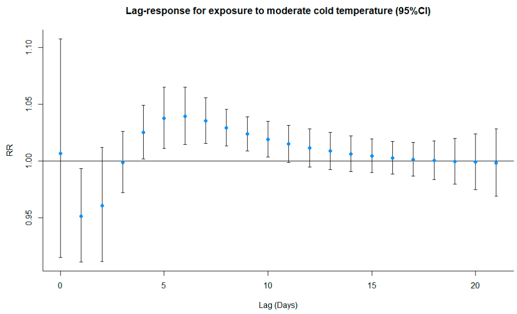

d) Females

Fig. S1 Lag-response curves for exposure to moderately cold temperatures (95% CI) for (a) the total population, (b) the elderly, (c) males, and (d) females.

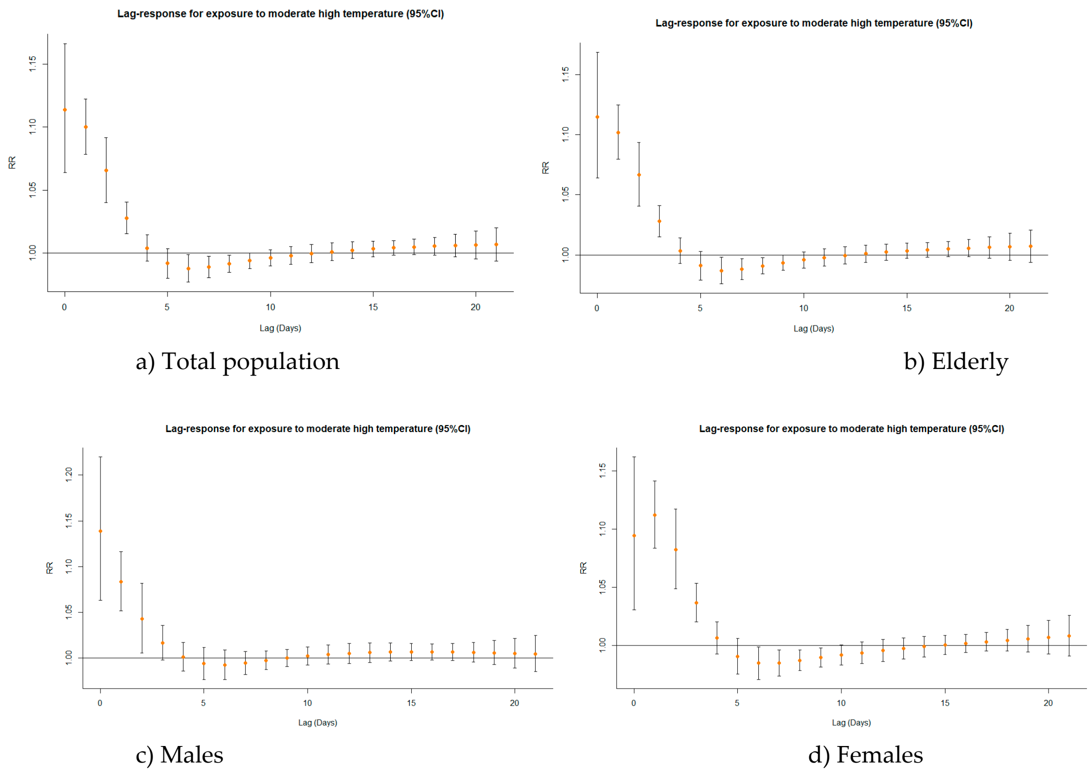

Fig. S2 Lag-response curves for exposure to moderately hot temperatures (95% CI) for (a) the total population, (b) the elderly, (c) males, and (d) females.

Table S2: The number of cardiorespiratory deaths (AN) attributed to air temperature and the fraction of cardiorespiratory mortality (AF) attributed to cold, hot, extremely cold and extremely hot conditions for the total population and its subgroups in EMT between 1999 and 2018.

	**Total number of deaths**	**AN**	**Extreme cold AF (95% eCI)**	**Extreme heat AF (95% eCI)**	**Moderate cold AF (95% eCI)**	**Moderate heat AF (95% eCI)**
Total population	72123	10035 (4847-14179)	0.90 (0.65-1.12)	0.70 (0.52-0.88)	7.61 (0.70-13.62)	4.96 (3.16-6.66)
Males	34526	4841 (1497-7418)	0.74 (0.40-1.05)	0.69 (0.45-0.95)	7.45 (-2.81-15.88)	5.38 (2.52-7.87)
Females	37597	5289 (1443 - 8641)	1.05 (0.69-1.37)	0.72 (0.46-0.97)	7.97 (-3.02-17.03)	4.60 (1.96-6.71)
Elderly (≥65 years old)	65173	9896 (5541-13783)	0.95 (0.70-1.18)	0.78 (0.59-0.96)	8.21 (1.23-14.73)	5.55 (3.66-7.41)
		Fraction of days* (%)	1.08	0.96	64.42	33.50

based on the total population MMT

Table S3: MMT and cumulative relative risks of cardiorespiratory mortality for various lag intervals, considering constant exposure, under different models for the total population and its subgroups in EMT between 1999 and 2018.

		qAIC	Minimum Mortality Temperature (MMT, °C)	Minimum Mortality Percentile (MMP)	Relative Risk for extreme cold (95% CI)	Relative Risk for extreme heat (95% CI)	Relative Risk for moderate cold (95% CI)	Relative Risk for moderate heat (95% CI)
Total population	Main	37507.46	20.9	65	1.74 (1.44-2.10)	1.82 (1.52-2.18)	1.19 (1.00-1.42)	1.33 (1.20-1.48)
	Df/year for seasonal control: 4	37592.63	22.8	72	1.75 (1.50-2.03)	1.30 (1.21-1.40)	1.22 (1.07-1.39)	1.11 (1.07-1.15)
	Df/year for seasonal control 9	37509.26	20.1	63	1.64 (1.36-1.97)	1.99 (1.63-2.41)	1.15 (0.97-1.37)	1.41 (1.25-1.59)
	Lag period: 7 days	37626.22	21.2	66	1.32 (1.19-1.47)	1.71 (1.55-1.89)	1.11 (1.01-1.22)	1.29 (1.21-1.36)
	Lag period: 14 days	37543	21.6	67	1.66 (1.43-1.92)	1.73 (1.52-1.98)	1.16 (1.01-1.33)	1.28 (1.19-1.38)
	df/RH: 5	37511.22	20.9	65	1.73 (1.44-2.09)	1.82 (1.52-2.18)	1.19 (1.00-1.42)	1.33 (1.20-1.48)
	No RH	37506.32	21.1	66	1.75 (1.45-2.11)	1.77 (1.48-2.11)	1.19 (1.00-1.42)	1.31 (1.18-1.45)
Males	Main	31813.83	20.5	64	1.60 (1.23-2.08)	1.88 (1.44-2.44)	1.20 (0.94-1.53)	1.36 (1.17-1.59)
	Df/year for seasonal control: 4	31839.05	22.6	71	1.66 (1.34-2.05)	1.27 (1.14-1.41)	1.16 (0.97-1.40)	1.10 (1.05-1.16)
	Df/year for seasonal control 9	31820.08	20	63	1.53 (1.17-2.00)	2.03 (1.54-2.69)	1.21 (0.95-1.55)	1.43 (1.20-1.71)
	Lag period: 7 days	31885.63	21.1	66	1.37 (1.18-1.59)	1.63 (1.41-1.88)	1.15 (1.01-1.32)	1.26 (1.16-1.36)
	Lag period: 14 days	31844.04	21	66	1.60 (1.30-1.96)	1.75 (1.44-2.12)	1.14 (0.95-1.38)	1.30 (1.16-1.46)
	df/RH: 5	31817.47	20.5	64	1.60 (1.23-2.08)	1.88 (1.44-2.45)	1.20 (0.94-1.53)	1.36 (1.17-1.59)
	No RH	31811.56	20.7	65	1.61 (1.24-2.10)	1.80 (1.39-2.33)	1.21 (0.94-1.55)	1.33 (1.15-1.55)
Females	Main	32622.82	21.5	67	1.88 (1.44-2.45)	1.78 (1.39-2.27)	1.19 (0.92-1.52)	1.30 (1.14-1.50)
	Df/year for seasonal control: 4	32610.8	23	73	1.83 (1.49-2.26)	1.32 (1.20-1.46)	1.28 (1.07-1.53)	1.12 (1.06-1.17)
	Df/year for seasonal control 9	32638.46	20.2	63	1.73 (1.33-2.24)	1.94 (1.48-2.54)	1.09 (0.86-1.39)	1.39 (1.17-1.64)
	Lag period: 7 days	32712.96	21.1	66	1.29 (1.11-1.49)	1.79 (1.56-2.05)	1.07 (0.94-1.22)	1.31 (1.21-1.42)
	Lag period: 14 days	32655.26	22.1	69	1.73 (1.40-2.13)	1.72 (1.44-2.06)	1.18 (0.98-1.43)	1.26 (1.15-1.39)
	df/RH: 5	32626.12	21.4	67	1.87 (1.43-2.44)	1.77 (1.39-2.27)	1.18 (0.92-1.52)	1.30 (1.13-1.49)
	No RH	32618.2	21.5	67	1.88 (1.44-2.45)	1.74 (1.37-2.22)	1.19 (0.92-1.52)	1.29 (1.13-1.47)
Elderly (≥65 years)	Main	36778.92	20.7	65	1.81 (1.49-2.20)	1.94 (1.60-2.36)	1.22 (1.02-1.47)	1.38 (1.23-1.54)

	Df/year for seasonal control: 4	36850.44	22.8	72	1.77 (1.51-2.07)	1.33 (1.23-1.44)	1.22 (1.06-1.40)	1.12 (1.08-1.16)
	Df/year for seasonal control 9	36784.25	19.9	62	1.69 (1.39-2.06)	2.12 (1.72-2.60)	1.19 (1.00-1.43)	1.46 (1.28-1.67)
	Lag period: 7 days	36896.11	21	66	1.34 (1.20-1.50)	1.80 (1.61-2.00)	1.11 (1.00-1.22)	1.32 (1.24-1.41)
	Lag period: 14 days	36814.53	21.4	67	1.69 (1.45-1.97)	1.83 (1.59-2.11)	1.16 (1.01-1.34)	1.32 (1.22-1.43)
	df/RH: 5	36782.74	20.7	65	1.80 (1.48-2.19)	1.94 (1.60-2.36)	1.22 (1.02-1.46)	1.38 (1.23-1.54)
	No RH	36777.75	20.8	65	1.81 (1.49-2.21)	1.89 (1.56-2.28)	1.22 (1.02-1.47)	1.36 (1.22-1.51)

Table S4: The number of cardiorespiratory deaths (AN) attributed to air temperature and the fraction of cardiorespiratory mortality (AF) attributed to cold, hot, extremely cold and extremely hot conditions under different models for the total population and its subgroups in EMT between 1999 and 2018.

		AN	Extreme cold AF (95% eCI)	Extreme heat AF (95% eCI)	Moderate cold AF (95% eCI)	Moderate heat AF (95% eCI)
Total population	Main	10035 (4847. -14179)	0.9 (0.65-1.12)	0.7 (0.52-0.88)	7.61 (0.70-13.62)	4.96 (3.16-6.66)
	Df/year for seasonal control: 4	9015 (5340-12339)	0.89 (0.69-1.09)	0.35 (0.26-0.42)	9.52 (4.08-14.54)	1.9 (1.25-2.51)
	Df/year for seasonal control 9	9428 (4863-13642)	0.81 (0.56-1.05)	0.78 (0.58-0.97)	5.76 (-1.16-11.75)	5.98 (3.64-8.12)
	Lag period: 7 days	8097 (5230-10725)	0.46 (0.31-0.59)	0.63 (0.53-0.72)	5.84 (1.45-9.82)	4.4 (3.44-5.40)
	Lag period: 14 days	9711 (5757-13369)	0.84 (0.65-1.02)	0. 65 (0.51-0.78)	7.89 (2.32-13.14)	4.29 (3.03-5.53)
	df/RH: 5	9982 (4950 -14009)	0.89 (0.66-1.11)	0.70 (0.50-0.88)	7.54 (0.00-14.21)	4.96 (3.19-6.64)
	No RH	9952 (4939 -14432)	0.90 (0.66-1.12)	0.67 (0.50-0.86)	7.78 (1.00-14.15)	4.69 (2.88-6.14)
Males	Main	4841 (1497 7418)	0.74 (0.40-1.05)	0.69 (0.45-0.95)	7.45 (-2.81-15.88)	5.38 (2.52-7.87)
	Df/year for seasonal control: 4	3483 (77 3-57589)	0.81 (0.52-1.07)	0.29 (0.17-0.40)	7.30 (-0.72-14.11)	1.80 (0.89-2.71)
	Df/year for seasonal control 9	5112 (1892-7634)	0.66 (0.30-0.96)	0.76 (0.48-1.01)	7.43 (-2.52-15.86)	6.22 (2.70-8.86)
	Lag period: 7 days	4108 (2163-5726)	0.48 (0.26-0.66)	0.54 (0.38-0.66)	6.96 (0.67-12.23)	4,00 (2.58-5.47)
	Lag period: 14 days	4198 (1580-6477)	0.76 (0.48-1.03)	0.61 (0.42 -0.79)	6.38 (-1.35-12.91)	4.58 (2.51-6.52)
	df/RH: 5	4821 (1519-7540)	0.73 (0.37-1.04)	0.69 (0.43-0.94)	7.38 (-3.06-15.90)	5.40 (2.55-7.98)
	No RH	4776 (1168-7489)	0.74 (0.42-1.05)	0.65 (0.39-0.90)	7.66 (-2.17-16.82)	5.00 (2.34-7.42)
Females	Main	5289 (1443-8641)	1.05 (0.69-1.37)	0.72 (0.46-0.97)	7.97 (-3.02-17.03)	4.60 (1.96-6.71)
	Df/year for seasonal control: 4	5499 (2863-7532)	0.96 (0.67-1.22)	0.41 (0.28-0.52)	11.46 (4.2-17.97)	1.99 (1.15-2.82)
	Df/year for seasonal control 9	493 (532 -7151)	0.95 (0.59-1.27)	0.80 (0.53-1.05)	4.18 (-6.64-12.95)	5.73 (2.76-8.60)
	Lag period: 7 days	414 (1974-5782)	0.44 (0.23-0.62)	0.72 (0.56-0.85)	4.88 (-0.96-10.41)	4.73 (3.27-6.03)
	Lag period: 14 days	5646 (2681-8327)	0.91 (0.63-1.15)	0.69 (0.48-0.86)	9.57 (0.54-17.19)	4.07 (2.41-5.56)

	df/RH: 5	5258 (1306-8392)	1.04 (0.69-1.36)	0.71 (0.45-0.95)	7.91 (-3.2-17.98)	4.59 (2.16-6.82)
	No RH	5267 (1287-8407)	1.05 (0.72-1.38)	0.70 (0.45-0.94)	8.11 (-2.58-16.84)	4.42 (1.98-6.63)
Elderly (≥65 years)	Main	9897 (5543-13783)	0.95 (0.70-1.18)	0.78 (0.59-0.96)	8.21 (1.23-14.73)	5.55 (3.66-7.41)
	Df/year for seasonal control: 4	8486 (4944-11735)	0.92 (0.71-1.13)	0.39 (0.30-0.47)	9.68 (4.61-15.14)	2.09 (1.45-2.73)
	Df/year for seasonal control 9	9584 (5300-13438)	0.85 (0.56-1.08)	0.86 (0.63-1.05)	6.72 (-0.04-13.03)	6.59 (4.24-8.77)
	Lag period: 7 days	7543 (4720-10115)	0.49 (0.33-0.62)	0.70 (0.58-0.79)	5.68 (1.50-9.96)	4.84 (3.73-5.82)
	Lag period: 14 days	9107 (5615-12407)	0.88 (0.67-1.06)	0.72 (0.56 0.85)	7.82 (1.53-13.10)	4.79 (3.48 -6.03)
	df/RH: 5	9850 (5335-13698)	0.95 (0.71-1.20)	0.78 (0.57-0.96)	8.14 (1.57-14.66)	5.56 (3.61-7.38)
	No RH	9812 (5446 -13869)	0.95 (0.70-1.17)	0.75 (0.57-0.94)	8.36 (0.29-15.06)	5.29 (3.49-7.00)

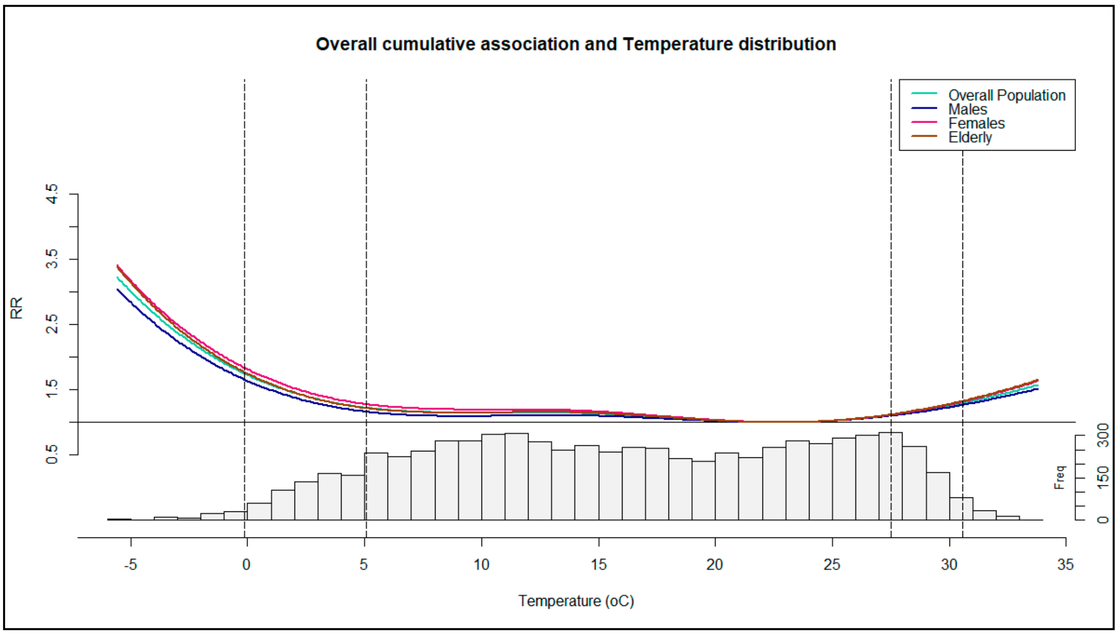

Fig. S3 The overall cumulative exposure-response curve of the mean daily temperature for the total population and its subgroups in EMT, for a lag period of 21 days with the related temperature distribution considering 4 Df/year for seasonal control.

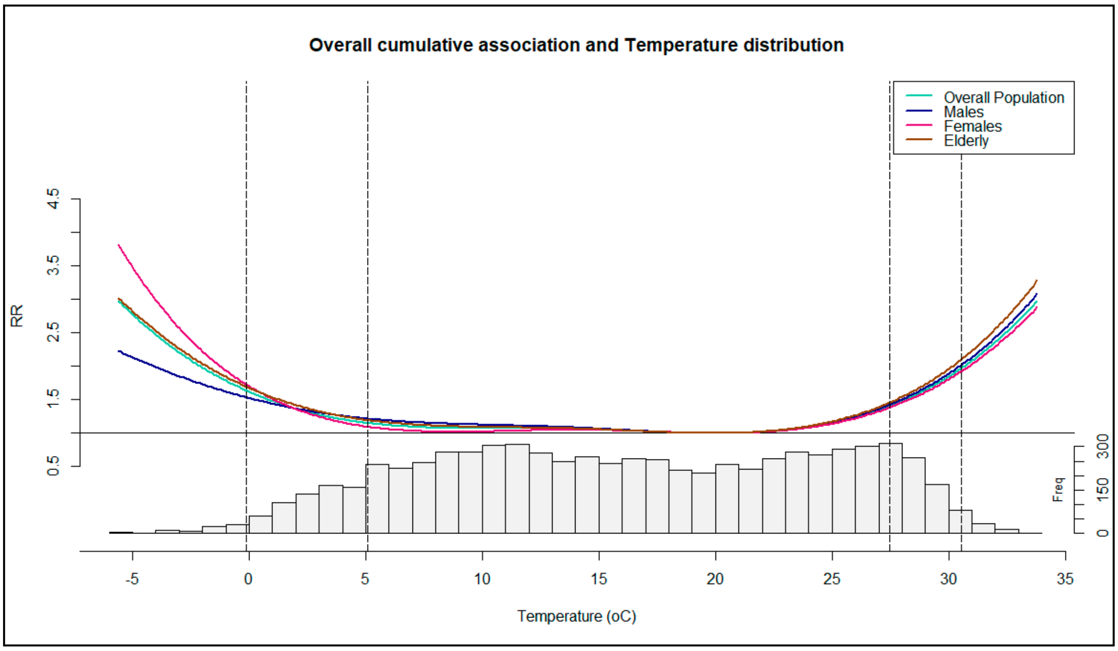

Fig. S4 The overall cumulative exposure-response curve of the mean daily temperature for the total population and its subgroups in EMT, for a lag period of 21 days with the related temperature distribution considering 9 Df/year for seasonal control.

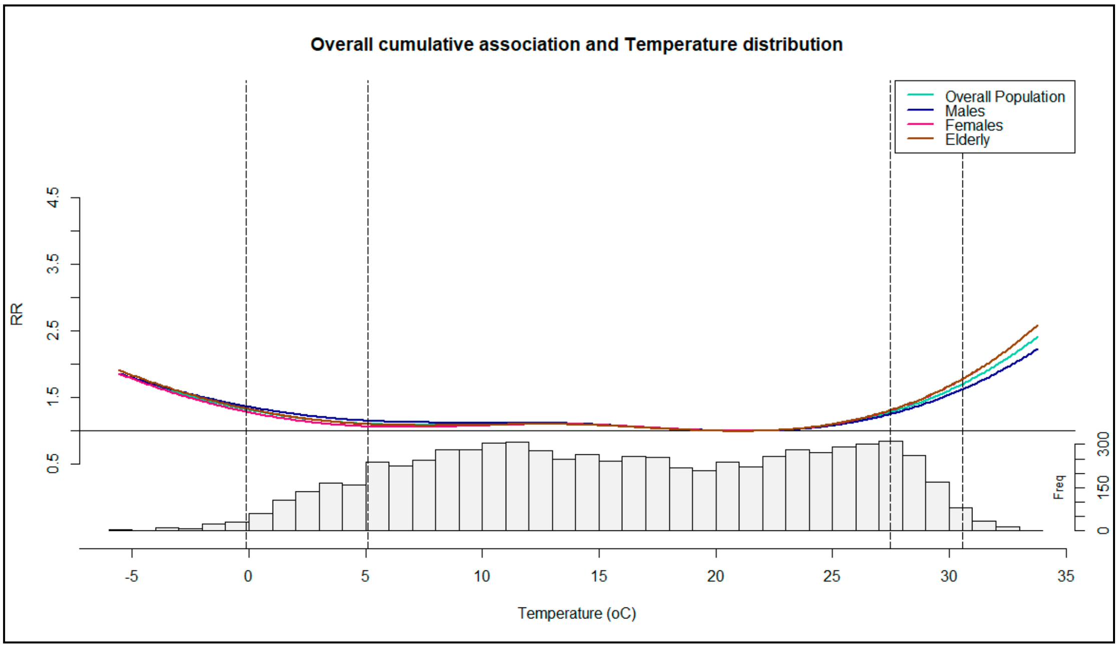

Fig. S5 The overall cumulative exposure-response curve of the mean daily temperature for the total population and its subgroups in EMT, for a lag period of 7 days with the related temperature distribution.

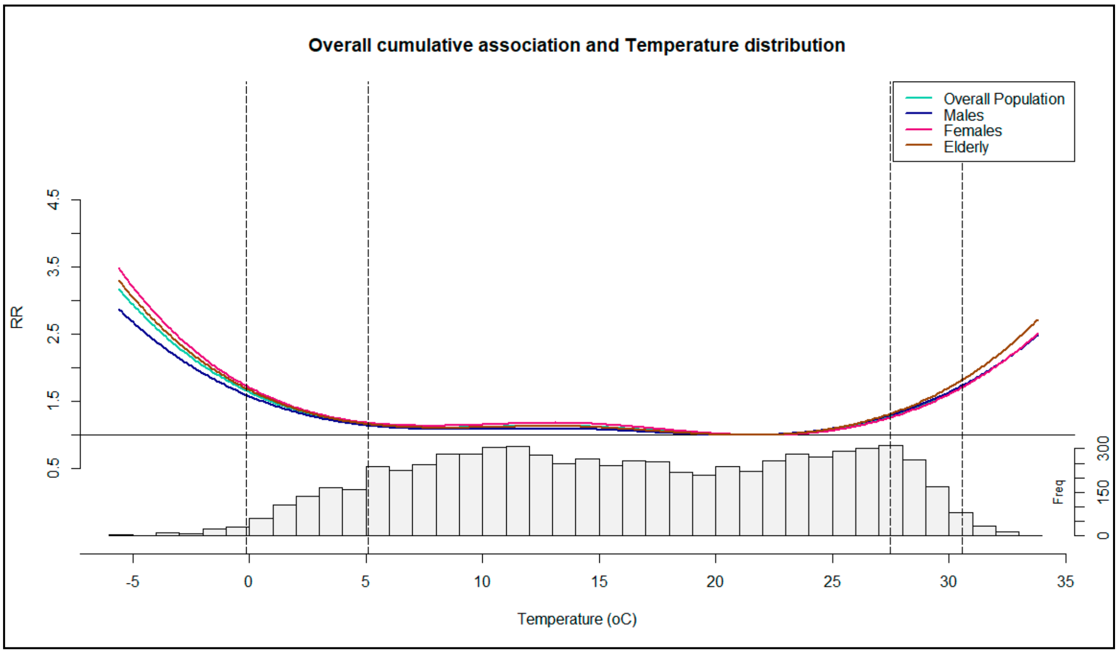

Fig. S6 The overall cumulative exposure-response curve of the mean daily temperature for the total population and its subgroups in EMT, for a lag period of 14 days with the related temperature distribution.

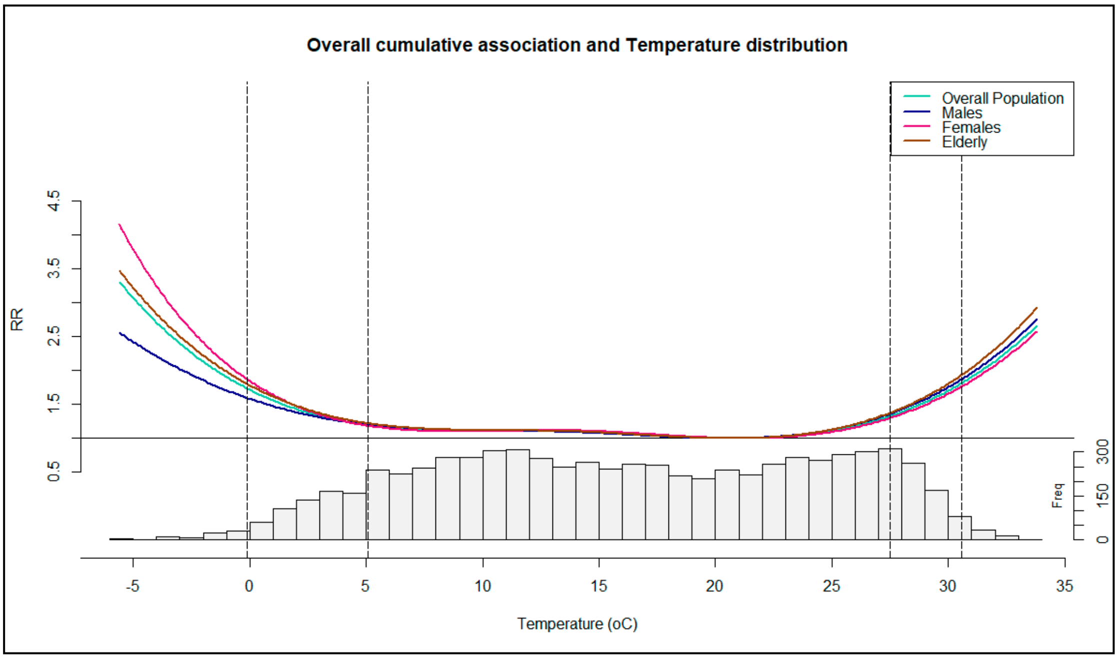

Fig. S7 The overall cumulative exposure-response curve of the mean daily temperature for the total population and its subgroups in EMT, for a lag period of 21 days with the related temperature distribution considering 5Df for relative humidity.

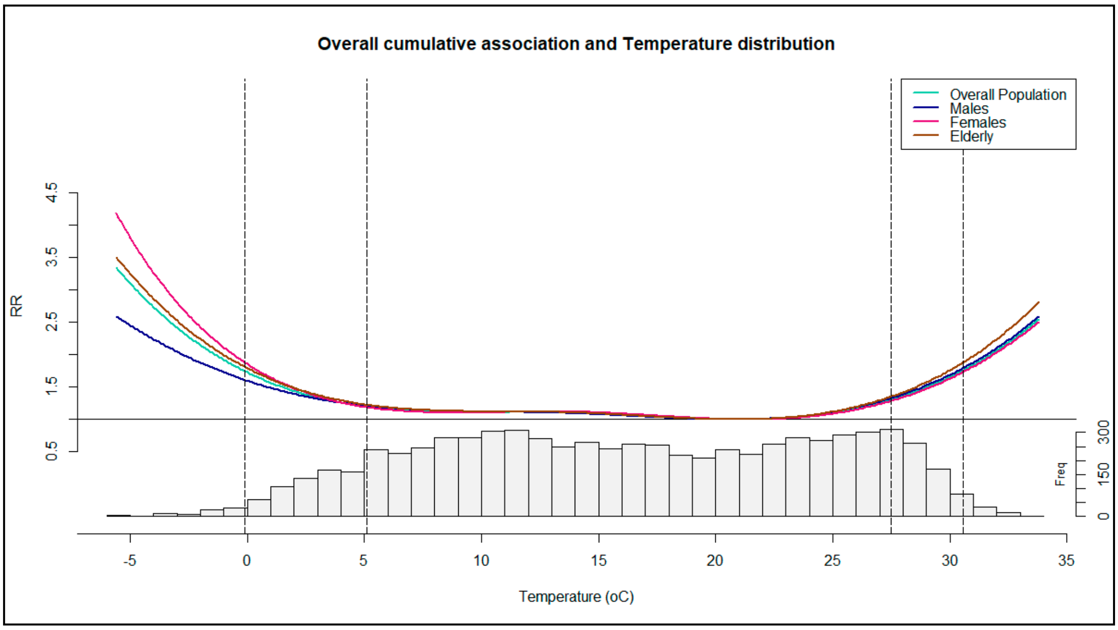

Fig. S8 The overall cumulative exposure-response curve of the mean daily temperature for the total population and its subgroups in EMT, for a lag period of 21 days with the related temperature distribution considering no relative humidity effect.
